# Mind-Stuff and Withdrawal of the Senses: Toward an Interpretation of Pratyahara in Contemporary Postural Yoga

**DOI:** 10.1177/13634593231222450

**Published:** 2024-02-04

**Authors:** Elizabeth McKibben

**Affiliations:** Te Herenga Waka – Victoria University of Wellington, New Zealand

**Keywords:** biomedicine, esthetics, health, neoliberalism, pratyahara, yoga

## Abstract

Yoga has become a popular health and wellbeing practice that draws on ancient philosophy. Pratyahara is a core tenet of yoga practice and is often translated to mean withdrawal of the senses. Withdrawing from the senses plays a key role in aiding yoga practitioners to find spiritual enlightenment by transcending the worldly. Withdrawing from the material world, however, does not neatly fit within the parameters of the contemporary postural yoga industry. This paper looks at the conceptual origins of pratyahara through stances relevant to health research. The author weaves biomedical, esthetic, and neoliberal onto-epistemological stances through health discourse to discuss how postural yoga both resists and replicates power imbalances. In so doing the author emphasizes the paradoxical nature of pratyahara as it is reflected in socio-political tensions of the yoga industry. To conclude, the author suggests that pratyahara itself can be useful in resolving this tension as yoga fulfills a philosophical prerogative for social change.

## Introduction

“Mind-stuff” is an English term used to describe the cluttering of consciousness with sensory stimuli. Adapted from the Sanskrit word “citta,”^1^ mind-stuff in contemporary parlance is a central tenet of yoga practice. The practice of yoga seeks to calm the mind by lessening the influence of sensory stimulation that results in mind-stuff. Withdrawing from the senses is thus a key part of yoga’s purpose: calming the fluctuations of the mind (“citta vṛtti nirodhah” in Sanskrit) (Patañjali, 2009, verse 1.2).

Calming the mind resonates with yoga’s contemporary application in wellbeing, yet this description emerges from ancient spiritual philosophy. Patanjali’s Yoga Sutras are a collection of spiritual aphorisms that have become standardized in yoga teaching curriculums across the globe (Yoga, 2023). Patanjali’s Yoga Sutras, as we know them today, include eight steps, referred to as “limbs.” These limbs guide readers through ethical, physical, and contemplative practices aimed at interrupting a karmic cycle of suffering. Patanjali considers spiritual suffering as the way in which humans are tethered to the physical world (Bryant, 2009). Through the limbs, Patanjali suggests easing suffering by relinquishing attachment to the material world.

Pratyahara, often translated as “withdrawal of the senses,” is the fifth limb of Patanjali’s Yoga Sutras. Pratyahara involves detaching from the distraction of external stimuli perceived through touch, taste, smell, sight, and hearing. Pratyahara instead draws awareness inwards. This internal focus is cultivated through a yoga practice that can help one move closer to spiritual liberation by renouncing the material world. Renouncing the material world was a key feature of the yogic lifestyle when Patanjali wrote the yoga sutras.^2^ During this time in pre-India, spiritual status was signified through ascetic life. Ascetics would lead nomadic lives, often living in isolation (Denton, 2004). While solitude and material renunciation were key features of yoga practice when the sutras were written, these practices have not necessarily transferred to modern postural yoga.

Yoga scholarship in the 20th and 21st century has debated the legitimacy of modern yoga and described it as a new practice that is related to, but distinct from, ancient yoga (De Michelis, 2020; Foxen, 2021; White, 2014). Scholars argue that 20th century western physical culture has influenced a re-interpretation of yoga that focuses on the body (Singleton, 2010). This influence has likewise grounded contemporary yoga in a secular-spiritual health discourse (De Michelis, 2020). Mindfulness and physical activity are woven together into a yoga practice that predominantly focuses on physical postures (asana). This emphasis on asana has shaped an industry that at times disregards the principles of yoga philosophy. For example, renowned 20th century yoga teacher Swami Sivananda believed that pratyahara is the essence of yoga (Frawley, 2014). Yet, more recently, pratyahara is also described as the “neglected limb of yoga” (Machado, 2022). Neglecting the essence of yoga in the study and practice of yoga begs the question, can contemporary yoga strive to fulfill the purposes of its ancient philosophy?

This paper develops a contemporary understanding of the concept of pratyahara by advancing our understanding of the practice beyond the binary of old versus new ways of doing yoga. It does so by exploring the conceptual origins of pratyahara and presents some ways in which scholars have attempted to operationalize pratyahara. It will identify how biomedicine, esthetics, and neoliberalism support and contradict the meaning of pratyahara, reflecting sociopolitical tensions of a yoga industry. To conclude, I will propose how pratyahara itself may be able to help navigate these tensions as mind-stuff becomes central in moving toward social change.

## Conceptual origins of Pratyahara

Contemporary explorations into pratyahara state that the esoteric significance of the concept is beyond the scope of health research (Vaishnav et al., 2022). Researchers have struggled to navigate the distinction between conceptualization and practice of this yogic technique (Pandi-Perumal et al., 2022). De-mystifying the conceptual origins of Pratyahara may help to better apply theoretical significance to the practice and study of yoga.

Patanjali’s Yoga Sutras draw upon Samkhya philosophy (Bronkhorst, 1985). This way of knowing suggests that the universe is constituted of dualistic elements: puruṣa (pure consciousness) and prakṛti (matter). Pure consciousness is a state of spiritual liberation, meanwhile matter is constituted of elements perceivable to the human senses. Engaging with the senses thus keeps people attached to the material (prakṛti). Humans both are and experience prakṛti through a sensual interaction of mind, body, and environment. Patanjali advocates cutting this sensual tether to the worldly through a yoga practice that transcends the body (Bryant, 2009). Pratyahara in particular provides a clear means of cutting this worldly tether by withdrawing the senses in order to calm the mind (“citta” in Sanskrit).

To access pure consciousness, there needs to be an optimal balance in the qualities of body, mind, and environment. The qualities of the material world (“gunas” in Sanskrit) are described as interacting elements of mind-stuff; sattva, rajas, and tamas (Woods, 1915). The gunas each have different characteristics; tamas is stagnant, rajas is transforming, and sattva is illuminating. Woods (1915) describes the disturbance of citta as instances when rajas and tamas override sattva. Imbalances between rajas, tamas, and sattva cause disturbances to the mind that impact one’s ability to transcend the material world. Taking in these forms of sensory stimulation leaves an imprint upon the mind that prevents spiritual transcendence. In order to understand how to withdraw from the material qualities of the mind, first we must understand how qualities of the material world make an imprint upon consciousness.

According to Patanjali, the material world makes an imprint upon citta through the senses (Bryant, 2009). The primary senses of sight, hearing, touch, taste, and smell create a bridge between material and spiritual worlds. There is a dynamic relationship between body and environment that can enable or inhibit rajasic, tamasic, and sattvic qualities from imprinting upon the mind. This interplay of external and internal navigates three key domains; sense objects, sense organs, and sense perception.

Sense objects are elements of the material world that produce sensory stimuli. Also understood as “entities of the outer world,” sense objects are partially responsible for establishing the content of the mind (Maas, 2010). The sense organs, those elements of the body that can see, hear, feel, smell, and taste, interpret sense objects such that they become representations in the mind. The representation of sense objects through sense organs forms sense perceptions that make up citta. The material qualities of sense objects, thus have the potential to make an imprint upon the mind by virtue of how the body interacts with the external world. This mind-stuff is an internal reflection of the forms, shapes, and social meanings that emerge from sensory experiences grounded in the material world. To further explain this phenomenon, I will turn to an analogy.

When one wears seeing eyeglasses, one may have a crisp and clear view of the street names on street signs. The signs have meaning and help to situate a person in their broader environment. Yet, when one does not wear seeing eyeglasses, the street names are unclear. Perhaps one could detect that there is in fact a street sign, but not the details on it that signal something about the environment. Without the glasses, some mind-stuff is kept at bay because the raw qualities of the sense object (the sign) have less significance than the social symbol of the sense object (the street name). Yet, the recognition of the street sign in the first place still produces mind-stuff. While detaching from all meaning attributed to sensory perception may be the ideal of a transcendental yoga practice, stymieing this process is the function of pratyahara.

While pratyahara is often translated into English to mean “withdrawal of the senses,” this translation is incomplete. The Sanskrit word is derived from a combination of two root words; “prati” and “ahara.” Both prati and ahara carry various meanings. Prati can mean away from or toward. Ahara can mean food, nourishment, gathering, or something taken in. When merged, pratyahara can suggest much more than what is characterized in the English translation as merely an elimination of sense experience.

Pratyahara has been uniquely operationalized and defined in various inquiries on the topic. Some scholars interpret pratyahara as a detachment from the external world (Vaishnav et al., 2022). Vaishnav et al. (2022) then elaborate on this definition by describing pratyahara as a focus on inner awareness. Inner awareness may imply spiritual consciousness but is not clearly distinguished from cognitive or body awareness, which are likewise elements of the material world. Other definitions suggest pratyahara is a lessening of the power of the senses over consciousness (Patil et al., 2018). These working operationalizations demonstrate distinctly nuanced understandings of the concept that highlight a paradox. While pratyahara can be understood as the “de-objectification of consciousness,” detachment from sense objects can only be assessed in direct contact with those sense objects (Raveh, 2015). This de-objectification of consciousness relies upon engaging with the material world. Unlike its translation as an absolute removal of sensory experience, pratyahara involves a more complex relationship between external and internal worlds that challenges its current operationalization in health research.

Patanjali’s Yoga Sutras introduce pratyahara as a core concept and practice of yoga. This complex and multi-faceted way of understanding how the senses connect the material and spiritual worlds is at times baffling, contradictory, and overall under-studied. Indeed, Patanjali’s Yoga Sutras are a convoluted text that have been interpreted and simplified into more palatable steps (White, 2014). Nevertheless, pratyahara presents a means of striving to calm the mind, a popular imperative in the yoga industry. Dissociating consciousness from the material world thus calls for a deeper investigation of sensory engagement in order to interpret the philosophy of yoga in contemporary contexts.

## Pratyahara through Contemporary Ontologies

Pratyahara is conceptually embedded in many yogic texts, yet the concept and practice has been little explored in recent scholarship. While withdrawal of the senses may be a neglected area of study, sensory engagements have sparked academic inquiry in a wide range of fields. While there are numerous ways to understand yoga from outside-in, this paper will explore perspectives salient to modern postural yoga: biomedicine, esthetics, and neoliberalism. Each of these stances in some ways align with pratyahara’s conceptual origins, and yet simultaneously fail to comprehensively engage with pratyahara’s onto-epistemology. Exploring these diverse perspectives may help untangle the paradoxes and tensions of the yoga industry.

### Biomedical mind-stuff

Body-centricity has formed a scaffolding through which to understand the philosophy of contemporary yoga practice. Over the last 125 years, the more esoteric practices of yoga have been overshadowed by a focus on the performance of the body. When Swami Vivekananda introduced Patanjali’s Yoga Sutras at the World’s Parliament of Religion in 1893, he did not intend for the physical postures of yoga to be the biggest take away (Alter, 2005). Yet, throughout the 20th century, the power of yoga postures for strengthening the body and mind became central in yoga’s transnational health discourse (Alter, 2007). Popular texts such as *Health and Hatha Yoga* (Śivānanda, 1985) and *Science of Yoga* (Swanson, 2019) frame the practice of yoga within a western medical framework, and bolster yoga’s legitimacy as a health practice (Strauss, 2002). These approaches to understanding yoga simultaneously revere yoga as a beneficial health practice, meanwhile contradicting the core spiritual and philosophical ontology of yoga.

Western medicine privileges measurable, human-centered, singular perspectives on physiological indications of health. An ontological grounding in a sense-able world paired with a singular approach at first glance does not quite fit with the holistic imperatives of pratyahara and yoga. Di Placido discusses this tension by considering hybrid forms of yoga, some of which focus on biomechanics results in bodies being scrutinized through medical gaze (Di Placido, 2020). This emphasis on the body reflects medical perspectives that privilege knowledge based on observation and measurement (Mol, 2002). Facts, numbers, and evidence based on visual assessment of health are highly situated in a material world. Critiques of medical models of health suggest a need to move beyond the siloes of body systems in favor of integrative approaches to health (McEwen, 2017). Yoga’s holistic understanding of the body-mind-environment thus provides a means to challenge and advance medical epistemologies, just as biomedicine may still have a place in making sense of pratyahara.

Pratyahara may provide unique opportunities for improving health, and thus optimizing the body in sensory discernment. A study that explores the impacts of yoga nidra suggests that pratyahara may be useful in improving health and wellbeing through a moderation of stress response (Vaishnav et al., 2022). Selective attention through somatosensory, viscerosensory, and chemosensory processes form the basis of a psychological framework for self-regulation though pratyahara (Gard et al., 2014). Bi-directional and integrative feedback networks of neurological control have the potential to moderate stress-response. In a clinical trial, specific exercises that encourage disengaging from sensory experiences were shown to aid in reducing anxiety (Gulia and Sreedharan, 2022). In yet another neuroscientific study, pratyahara meditation was shown to activate the brain differently than other relaxation techniques (Fox et al., 2016). Research grounded in biomedical understandings of the body and mind demonstrate how pratyahara has the potential to influence physiological measures of health. Measuring pratyahara in this way may help legitimize pratyahara as a health practice. However, the biomedical mechanisms of pratyahara remain unclear in recent literature.

In biomedical accounts, the focus on an empirical reality prevents research from reconciling the paradoxical nature of pratyahara. In one account, sense experiences of the external world increase through pratyahara (Sharpe et al., 2021). Sharpe et al. (2021) detail how cognitive awareness of the external world increases as attention is diverted away from the body. In another account, pratyahara shifts attention to the body through interoception, and thus away from the environment (Gerbarg and Brown, 2015). In both accounts the body serves as a stark boundary between internal and external worlds by either being the point of attention, or the point of non-attention. This tension between the “truth” of pratyahara can be seen through conflicting understandings of the mechanisms of pratyahara that result in health benefits but fail to capture the polyvalence of the practice.

Biomedical perspectives may be necessary but incomplete ways of understanding pratyahara. In particular, biomedicine is useful for interpreting the impact of pratyahara on the body. This aligns with perspectives in religious philosophy that consider the withdrawal from the external world as only being measurable through direct engagement with the external world (Raveh, 2015). Engaging with this element of pratyahara thus provides a useful framework for understanding ways of optimizing the body for health through yoga practice. Yoga practices and research that focus on the biomedical changes in the body may indeed optimize sense organs so they may be better able to discern sattvic elements of sense objects. Yet, this understanding focuses exclusively on sense organs (the body) and does not epistemologically accommodate the entirety of sense objects, sense perception, and mind-stuff. To engage with yoga as a health practice, according to pratyahara, one must navigate both the interactions of body-mind-environment, and a singular awareness of body, mind, *and* environment. Medicalizing pratyahara may have utility for bolstering health and wellbeing but cannot be applied in isolation when trying to understand a concept that seeks to withdraw from a sense-able world.

### Esthetic mind-stuff

Looking toward diverse theory may provide additional insights into understanding the calming of mind-stuff through pratyahara. While pratyahara is concerned with the senses to withdraw from them, esthetics is concerned with understanding the world through the senses (Santayana, 1896). While esthetics may be most associated with the arts, they are likewise an important element of understanding health. For example, esthetically pleasing natural environments stimulate dopamine responses in the brain (Kirsch et al., 2016). Neurobiological, and cognitive explorations of esthetics pinpoint relationships between sense perception and emotion (Lopes, 2018). Lopes (2018) attempts to use affect to integrate esthetic theory with esthetic science, asserting that while these perspectives are conflicting, they are not mutually exclusive. Pratyahara may add yet another lens through which to navigate conflicting, yet complimentary conceptualizations of esthetic engagements as mind-stuff.

Philosophical interpretations of the senses range greatly through time and space. Aristotle considered the assimilation of sense perception and sense object as a virtuous trait that defines a developed mind (Lorenz, 2007). Dewey makes claims that engaging with sensory experiences is essential for leading a meaningful life (Haskins, 2019). Both of these perspectives, however distanced from one another in space and time, draw on a moral element of the senses, in which being “good” and living “well” are predicated upon an appropriate engagement with the material world.

While esthetics in the 20th century have been applied to the study of beauty in the visual and performing arts, the foundation of contemporary esthetic theorizing is situated in the body. In the 1700s, Baumgarten’s seminal work inquired into the ways that the senses traverse the boundaries of body, mind, and environment (Hammermeister, 2002). Similarly, the conceptual foundation of pratyahara places sensory experiences in a type of body-logic. Locating sensory experience in the body has been described as “somaesthetics,” which posits that sensory experiences are embodied interactions of internal and external worlds (Shusterman, 2012). Somaesthetics begin to merge a gap between body-logic and the environment that mirrors the dynamic between sense objects, sense organs, and sense perception.

Esthetic theories may enable a more holistic view of health, pratyahara, and the senses. In a philosophical take on the politics of sensation, Panagia discusses the senses as “moments of interruption” (Panagia, 2009). What Panagia means is that sense objects in space have the potential to re-configure expectations of sensory experience (Dickinson, 2018). The social meanings attributed to objects in space can be challenged by unexpected sensations. On the other hand, sensory experience may likewise affirm pre-existing beliefs about the purpose and utility of space and objects in them. In explorations of esthetics in religious space, the senses serve both as a boundary marker and as a means of blurring boundaries. In this way, engagement with sense objects from the external world is a process of embodiment (Brent Plate, 2012). Embodied sensory experience thus creates a perimeter around which sense perceptions define the socially constructed meanings of space. In other words, esthetic experiences from the external world fill the space of the body which then delineates social and sacred space as both of sense organs and sense objects. However, this understanding of esthetics as entangled in the body-mind-environment remains focused on sensory engagements situated in the body, which is also an element of the material world.

Posthuman esthetics provide yet another insight into the paradoxical relationship between immersion in, and liberation from, the material world. Existential posthumanism positions yoga as a natural progression from philosophical posthumanism (Banerji, 2021). Posthuman theory provides a framework for understanding the agency of the material world in esthetic engagements that compliments yoga’s ontology. Within this framework, the materiality of the world is the focal point for meaning-making relationships (Barad, 2007). Similarly, Jane Bennet’s work “Vibrant Matter” advocates for a re-conceptualization of “things” into “beings” with agentic possibilities (Bennett, 2010). Likewise, the gunastic properties of sense objects are also agencies; sattva is illuminating, rajas is transforming, and tamas is stagnant. These esthetic qualities are agencies in and of themselves, thus demonstrating how yogic philosophy positions agentic power, and meaning-making possibility in material objects similarly to posthuman theory.

The prerogative to withdraw from those sensory experiences through pratyahara, however, challenges posthuman theorizing. When applied directly to yoga, feminist new materialisms suggests that sensory experiences are profoundly entangled in the relationships between affect, bodies, and space (Jeffrey et al., 2021). In this work, the sensory-affective-corporeal-material is considered analogous to the state of spiritual union that yoga seeks to achieve. While this intermingling of body-mind-environment supports the ontological grounding of pratyahara, it is less clear how one practices withdrawal from the material world in an onto-epistemology that prioritizes the agency of the material world.

Esthetic experiences attributed to the theory and practice of yoga promote an industry that promises health and wellbeing benefits. Portraying a peaceful, calm, harmonious esthetic has become key in visual marketing strategies for yoga retreats in India (Puustinen and Rautaniemi, 2015). Indeed, yoga tourism relies upon this esthetic to seduce audiences with the promise of wellbeing (Telej and Gamble, 2019). The sensory experiences of yoga spaces also play a role in measuring the efficacy of yoga. In one clinical trial on pratyahara, the precise room temperature and humidity level played a role in yoga practitioners’ ability to “go deeper” into meditation (Sharpe et al., 2021). These environmental conditions curate an ideal sensory experience that has come to be associated with yogic prerogatives in a contemporary health and wellbeing industry. Esthetic preoccupation enables a neoliberal economy to profit from concepts of self-care and self-improvement that are embedded in texts like Patanjali’s Yoga Sutras.

### Neoliberal mind-stuff

Patanjali’s Yoga Sūtras have been translated and interpreted through key characters only within the last 150 years. Swami Vivekananda in particular is credited with a global distribution of the sutras. While he asserts a claim to the ancient philosophy of the sutras, his interpretation is communicated as an ideological commitment to the contemporary world (Madaio, 2017). As Madaio explains, Vivekananda adopted what he thinks is “good” about an eclectic smattering of theology and presented them as the eight limbs of yoga (Madaio, 2017). Patanjali’s Yoga Sutras were translated, interpreted, and then re-structured to fit into a more consumable form for western audiences. The eight limbs of yoga were then positioned in the minds of westerners as ancient ideology, despite only being re-constructed in the early 1900s (White, 2014). Since then, the lessons of the sutras have been widely communicated through a neoliberal philosophy that emphasizes self-control and personal development.

The promise of individually driven self-development is evident in the way the sutras provide a framework for pursuing a prescribed form of spiritual liberation. Health through yoga can be achieved by following a series of sequential, progressive steps. This form of a “how to” guide exerts power over prospective yoga practitioners by placing responsibility in individuals’ hands to follow a prescriptive, “correct,” yoga procedure. This precision of yoga practice can be seen in feminist explorations of Iyengar yoga, which critique the ways this lineage places female yoga practitioners in a separate corner of the classroom when they are menstruating (Wittich, 2018). Wittich (2018) speaks of a rigid alignment that exercises a hierarchy of power and control through strict physical forms. Enforcing these protocols as “good” spiritual practice diverts people away from the insidious and contradictory emphasis on socio-material power in the postural yoga industry.

Agency and power continue to appear as key features of yoga practice and research. In particular, the rise of yoga industry in the 20th century draws on political philosophies that exert power over. Neoliberal concepts such as “self-care” and “self-love” are often associated with yoga and mask neoliberal imperatives for health (Jain, 2020). De Michelis addresses “healthism” as a prominent discourse of contemporary yoga (De Michelis, 2020). Healthism refers to the influence of neoliberalism on creating individualistic, moral imperatives to pursue health (Crawford, 1980). In this frame, it is up to individuals to strive to adhere to normative standards of health. Such neoliberal ideals place power in the people and frameworks that dictate “correct” ways of practicing yoga. In turn, this ignores the ways that access to those resources is rooted in social factors such as race, class, and gender.

While yoga is often seen as an inclusive health practice, contemporary postural yoga operates through political frames that maintain a hierarchical status quo. Lean, toned, young, white, female bodies performing gymnastic-like shapes are most commonly depicted in popular media as “yoga bodies” (Hinz et al., 2021a, 2021b; Webb et al., 2017). These esthetic criteria prioritize normative identities of femininity, affluence, and whiteness (Bhalla and Moscowitz, 2019). Indeed, the yoga industry uses claims about health and wellbeing to “tame” women into literal shapes that patriarchal powers deem they should be able to fit into (Strings et al., 2019). An esthetic of health in the contemporary yoga industry reiterates mechanisms of control that are dubiously and often invisibly situated in yoga philosophy itself.

Hierarchies and discernment are embedded in the practice of pratyahara. In one definition, scholars suggest that pratyahara is in and of itself “control of the senses”(Sengupta, 2022). Control of the senses implies a moral discernment around which senses *should* be controlled. In pre-India, pratyahara was seen as a cure for mental illness due to its ability to help people detach from “undesirable thoughts”(Abhyankar, 2015). The term “undesirable thoughts” suggests a social or political force that discerns which thoughts are desirable, which are not, and for whom. Pratyahara, as understood through Patanjali’s Yoga Sutras, then likewise presents a mechanism for controlling the populous through an idealized form of spiritual engagement.

The yoga industry can likewise be seen as perpetuating an idealized esthetic of spirituality through a complicated entanglement of socio-material-consumer imperatives. Specifically, the yoga industry relies upon the esthetic of non-human materials to sell wellbeing (Jain, 2014). For example, Lululemon has a thriving company that sells “yoga pants” designed specifically for the practice of yoga. They suggest that an individual should buy material goods in order to be healthy, and in order to adequately practice yoga (Lavrence and Lozanski, 2014; Stokes, 2008). While pratyahara urges a relinquishing of the material world, the industry of yoga inherently ties yoga practitioners into the material through consumption in the name of self-care. Buying into specific curated material goods, and environmental experiences ironically perpetuates an engagement with an esthetic, sensually engaged practice of yoga. Pratyahara, as a process of withdrawing from those material elements, thus threatens an industry status quo.

## Pratyahara as resistance?

In the early 1900s, Mahatma Gandhi drew on pratyahara in pursuit of social change. In resistance to caste separation, Gandhi’s famous hunger strike involved a profound withdrawal from “ahara” (nourishment). Gandhi’s demonstration of yogic self-control spurred collective action against oppressive socio-material forces (Godrej, 2017). Some suggest that yoga has a “quintessential political expression” that can resist oppressive power (Kale and Novetzke, 2021). Yet, the actual capacity of postural yoga to make social change remains unclear (Oh and Sarkisian, 2012). Below, I consider how pratyahara may contribute to social change.

### Individual healing

Pratyahara’s prerogative to withdraw from the material world fits in with arguments of spiritual individualism. Proponents of spiritual individualism suggest that spiritual pursuits in contemporary contexts are dissociated from collective change, favoring well-being and self-transformation (Bellah and Bellah, 1996; Possamai, 2005). Self-transformation, transcendence, relaxation and escape are themes that capture the “life-changing” experiences of traveling to India for intensive yoga retreats (Dillette et al., 2019; Voigt et al., 2011). Withdrawing from day to day life by investing in retreats places the pursuit of wellbeing in a geographically and temporally located site, which remains distinct from “normal” life (Putcha, 2020a). These individualistic pursuits re-exert inequity by privileging affluence and distinguishing well-being from daily contexts and practices. Yet, pratyahara may still operate at the individual level to promote healing that indirectly benefits movements for social change.

Sites of yoga practice, and the practice itself, are often considered secular sanctuaries (Telles and Singh, 2017). These sanctuaries, literal places and also places within the self, provide a needed refuge from discrimination. In her autoethnographic account, Myers (2017) presents her yoga practice in a studio as an essential safe space for queerness. She recounts how yoga practice enables a finding of refuge within herself, and thus a means of escaping blatant homophobia in the outside world (Myers, 2017). Similarly, Van Hester discusses the role of yoga at home during the COVID-19 pandemic (VanHester, 2021). Her “Tale of the Fat, Beautiful, Black Butterfly” does not explicitly address pratyahara, but points to the value of withdrawal from external sensory stimuli to turn inwards and find refuge in an empowering “self-space.” Withdrawing from the outside world may be an essential tool for the wellbeing of people who face marginalization in their day to day lives. In this sense, pratyahara serves social justice prerogatives through its individual healing capacity.

Furthermore, pratyahara enables individuals to find healing from pain. In a study on the physiology and cognition of pratyahara meditation, researchers identified how detached awareness of the experience of pain decreased participants subjective experience of pain (Vallath, 2010). Through pratyahara, pain is re-framed by accessing an inner self-awareness that is a “witness” of pain in the material body (Vallath, 2010). Pain, a form of suffering in the material world, can thus be transcended through an individual practice. In studying pratyahara through paradigms which privilege the sense-able world, mainstream approaches to medicine legitimize yoga in the eyes of the status quo. So doing integrates holistic concepts of healing into neoliberal health care systems, which may open opportunities to witness the system with critical gaze. Evidence of pratyahara’s efficacy at the individual level thus serve pursuits of systemic change.

### Socio-political awareness

Pratyahara’s paradoxical engagement with and withdrawal from the material world present a challenging negotiation of avoidance and awareness of hierarchical norms. Translations such as “remov[ing] oneself from seeing, tasting, hearing, touching, and smelling” (Wilber, 2020) can easily be interpreted as removing oneself from seeing injustice, and thus engaging in spiritual bypassing. The term “spiritual bypassing” refers to utilizing spiritual practices like yoga to avoid psychological pain, trauma, and tension (Welwood, 1984). In a discussion of “International Yoga Day,” Lakshmi (2020) elaborates on how yoga choreographs discourses of health, peace, and harmony in order to obfuscate socio-political violence (Lakshmi, 2020). If pratyahara is to be useful in prompting social change, attending to awareness of the material world is of the utmost importance.

The people, places, and things in much of the mainstream yoga industry are rife with sensory stimuli that replicate harmful discourses. For example, the use of culturally appropriative music and imagery in yoga studios (Putcha, 2020b). In an exploration of white public space, authors reflect on the impacts of colonial artifacts while practicing yoga in museums (Strauss, 2017). Since sense-objects have embodied impact, the socio-political meanings of these objects produce sensory engagements that discursively deliver colonialism and oppression. Similarly, narrative accounts describe the prevalence of whiteness in yoga spaces as incompatible with the needs of women of color who often face racism in yoga studios (Nousheen and González Madrigal, 2021). Despite intentions to increase the accessibility of yoga by practicing in public spaces, these initiatives often remain ignorant of socio-political contexts and fail to tap into broader campaigns for social justice (Di Placido and Pedrini, 2022). Garnering awareness of such power-full exertions may be a first step in instigating change. Like with individual healing, identifying “pain” as produced in the socio-material world enables a detached witnessing of these events that prevents being consumed by oppression. As such, emphasizing an engagement with the senses as they produce and reproduce power dynamics may be able to instigate action against harmful spaces and practices.

Actively engaging with the material world and its production of power configures an awareness of and capacity to act upon marginalization in yoga spaces (Mangiarotti, 2021). Critical Yoga Studies actively engages with such a prerogative. Works such as *Yoga, The Body, and Embodied Social Change* apply intersectional feminism to visibilize the insidious mechanisms of the yoga industry (Berila et al., 2016). Similarly, transcending oppression through yoga has sparked important works such as *Practicing Yoga As Resistance: Voices of Color in Search for Freedom* (Hagan, 2021). This work taps into yoga’s potential for empowerment as a practice, as a field of intellectual inquiry, and its potential to transcend a disempowering industry. In this way, the engagement with the senses by seeing and celebrating diverse yoga practices is an important part of challenging the status quo of the postural yoga industry. Deliberately engaging with socio-political mind-stuff enables us to re-think the moral discernments about which social and political content should be engaged with and withdrawn from.

### Critical practice

Pratyahara involves a re-framing of the material world, including objects, the body, and thoughts themselves. Hewitson considers pratyahara’s “de-objectified” stance as essential in inquiry, which “should begin with the subject’s consciousness stripped of psychological and cultural content” (Hewitson, 2014). Yet pratyahara’s utility in making change relies upon the consciousness first being cluttered. In a deliberate, focused awareness of the socio-political-material contexts of yoga practice, individuals and collectives under-go a process of lessening the control of these forms of thinking over consciousness. In this way, pratyahara prompts a withdrawal from dominant ways of thinking about the world. The withdrawal from thought as an element of the material world may then suggest that there should likewise be a withdrawal from the very concept of pratyahara itself. In this vein, yoga provides a means of resisting intellectual and social replications of power imbalance that impose a “correct” way of understanding the world.

Pratyahara is thus a framework for approaching critical thinking and practice. Medical anthropologist and yoga researcher Joseph Alter explains that understanding yoga through yoga philosophy only results in intellectual tangles that perpetuate tautological contradictions (Alter, 2023). Interpreting pratyahara through biomedical, esthetic, and neoliberal contexts of the 20th and 21st century present an ontologically and epistemologically intersectional process of understanding meaning making through the engagement with, and disengagement from mind-stuff. Stepping outside of familiar ontological groundings provides a means of embracing the concept of pratyahara in processes of concept-making. Pratyahara thus forms a platform for drawing on, and withdrawing from many forms of understanding, epistemes, and ontologies to relinquish the power of a uni-lateral perspectives in explorations of health.

In conclusion, pratyahara presents a conceptually rich and complex understanding of the various paradoxes of the yoga industry. While the ancient practice of pratyahara as worldly renunciation favors an individualistic withdrawal from social dilemmas, it’s conceptual origins also provide tools for social change in contemporary contexts. Sensory withdrawal can promote healing and empowerment. Meanwhile, sensory engagements promote social awareness and moral discernment. The internal and external interact such that the material world holds less power over the embodied, affective self. Indirectly, this alters broader systems of health care and healing by promoting holistic wellbeing. Directly, this enables a greater capacity to respond to and act upon injustices. Withdrawing from the replication of power cannot be an absolute withdrawal of the senses, but rather, an active engagement with and recognition of the mind-stuff that perpetuates inequity. By engaging with the biomedical, esthetic, and transcendental dimensions of pratyahara, the yoga industry may be able to challenge power imbalances, promote healing, and navigate the complex landscapes of the yoga industry. The mind-stuff cultivated in pondering pratyahara is an attempt to engage with the material world in the hopes of harnessing the transformative power of yoga for equity in health and wellbeing.

## Notes

1. “citta” is often translated to “mind” in Sanskrit, and refers to all related states of consciousness, sub-consciousness, and unconsciousness.

2. It is speculated that Pātañjali’s Yoga Sūtras were written around 250 CE (Singleton, 2010).
